# Automated Organ Segmentation for Radiation Therapy: A Comparative Analysis of AI-Based Tools Versus Manual Contouring in Korean Cancer Patients

**DOI:** 10.3390/cancers16213670

**Published:** 2024-10-30

**Authors:** Seo Hee Choi, Jong Won Park, Yeona Cho, Gowoon Yang, Hong In Yoon

**Affiliations:** 1 Department of Radiation Oncology, Yonsei Cancer Center, Heavy Ion Therapy Research Institute, Yonsei University College of Medicine, Seoul 03722, Republic of Korea; clickby_s@yuhs.ac (S.H.C.);; 2 Department of Radiation Oncology, Gangnam Severance Hospital, Yonsei University College of Medicine, Seoul 06273, Republic of Korea; 3 Department of Radiation Oncology, Cha University Ilsan Cha Hospital, Cha University School of Medicine, Goyang 10414, Republic of Korea

**Keywords:** auto-segmentation, radiation therapy, OncoStudio, Protégé AI, IMRT, Korean patients, organs at risk

## Abstract

Background: Accurate delineation of tumors and organs at risk (OARs) is crucial for intensity-modulated radiation therapy. This study aimed to evaluate the performance of OncoStudio, an AI-based auto-segmentation tool developed for Korean patients, compared with Protégé AI, a globally developed tool that uses data from Korean cancer patients. Methods: A retrospective analysis of 1200 Korean cancer patients treated with radiotherapy was conducted. Auto-contours generated via OncoStudio and Protégé AI were compared with manual contours across the head and neck and thoracic, abdominal, and pelvic organs. Accuracy was assessed using the Dice similarity coefficient (DSC), mean surface distance (MSD), and 95% Hausdorff distance (HD). Feedback was obtained from 10 participants, including radiation oncologists, residents, and radiation therapists, via an online survey with a Turing test component. Results: OncoStudio outperformed Protégé AI in 85% of the evaluated OARs (*p* < 0.001). For head and neck organs, OncoStudio achieved a similar DSC (0.70 vs. 0.70, *p* = 0.637) but significantly lower MSD and 95% HD values (*p* < 0.001). In thoracic organs, OncoStudio performed excellently in 90% of cases, with a significantly greater DSC (male: 0.87 vs. 0.82, *p* < 0.001; female: 0.95 vs. 0.87, *p* < 0.001). OncoStudio also demonstrated superior accuracy in abdominal (DSC 0.88 vs. 0.81, *p* < 0.001) and pelvic organs (male: DSC 0.95 vs. 0.85, *p* < 0.001; female: DSC 0.82 vs. 0.73, *p* < 0.001). Clinicians favored OncoStudio in 70% of cases, with 90% endorsing its clinical suitability for Korean patients. Conclusions: OncoStudio, which is tailored for Korean patients, demonstrated superior segmentation accuracy across multiple anatomical regions, suggesting its suitability for radiotherapy planning in this population.

## Simple Summary

In this study, we compared two artificial intelligence (AI) tools, OncoStudio and Protégé AI, for their ability to automatically segment organs in Korean cancer patients undergoing radiation therapy. Manual contouring of organs is time-consuming and can vary among clinicians, potentially impacting treatment accuracy. We aimed to evaluate how well the aforementioned AI tools can assist clinicians by speeding up this process and ensuring precision. Our findings showed that OncoStudio, which was specifically developed for Korean patients, performed better in most cases than Protégé AI, a globally developed tool. These results suggest that population-specific AI tools can improve radiation therapy planning, ultimately enhancing patient outcomes and reducing clinician workload.

## 1. Introduction

Radiation therapy, a cornerstone of cancer treatment, has advanced significantly with the adoption of intensity-modulated radiation therapy (IMRT) [1,2]. In IMRT, the precise contouring of tumors and surrounding organs at risk (OARs) is crucial for developing an optimal treatment plan that maximizes radiation delivery to the tumor while minimizing exposure to healthy tissues. However, the manual contouring process is labor-intensive and requires substantial time and specialized expertise from clinicians. This process involves the meticulous delineation of multiple anatomical structures across various imaging modalities, such as computed tomography (CT) and magnetic resonance imaging (MRI), which can introduce variability depending on the clinician’s experience [3,4,5]. Therefore, IMRT planning demands considerably more effort than traditional radiotherapy techniques, increasing the workload and the potential for inconsistencies, which may contribute to suboptimal treatment outcomes.

Automated segmentation using deep learning has emerged as a transformative solution to the challenges of manual contouring in radiation oncology, offering improved workflow efficiency, reduced interobserver variability, and enhanced overall accuracy [6,7,8,9,10,11]. Compared with traditional atlas-based methods, deep learning methods, particularly convolutional neural networks, have shown superior performance in delineating tumors and OARs [12,13,14]. Since the late 2010s, research in this field has expanded, with a stronger emphasis on organ segmentation rather than tumor segmentation [12]. Recent studies have demonstrated the effectiveness of AI tools in improving the accuracy of segmenting complex anatomical structures, such as the head and neck region, and in adapting to different anatomical variations across patient populations [12]. For example, atlas-based auto-segmentation in the head and neck region significantly reduced manual workload [15], and deep learning-based methods showed superior accuracy, particularly in complex anatomical regions, like the thorax [16]. Notably, there have been significant advancements in breast cancer target delineation, along with successful applications of synthetic contrast CT for cardiac structures and brain metastasis segmentation [17,18,19]. These studies underscore the continuous evolution of AI tools in radiation therapy, with each advancement addressing specific anatomical and clinical challenges.

However, studies predominantly use small datasets, prioritizing the development of new models over evaluating existing methods or their clinical applicability. This has raised concerns about overfitting, especially in rare or atypical cases, and has highlighted the limitations of applying a single auto-segmentation model uniformly across diverse patient populations or disease types, thereby hindering its widespread adoption in clinical practice.

This study aims to compare the performance and clinical applicability of two artificial intelligence (AI)-based automated segmentation tools, developed using deep learning techniques, specifically for Korean cancer patients. Manual contours created by radiation oncologists were compared to auto-contours using various mathematical comparison metrics, across 1200 cases involving multiple anatomical regions, to assess the strengths and weaknesses of each tool. Expert feedback, including input from radiation oncologists, residents, and radiation therapists, was gathered through online surveys to evaluate the usability and clinical feasibility of these tools. The study analyzes which AI tool achieves higher concordance with manual contours by anatomical site and is more likely to be preferred in clinical practice. The novelty of this research lies in its large-scale, multi-organ evaluation combined with direct clinical feedback, providing a comprehensive assessment of both technical performance and real-world applicability. Ultimately, this study aims to identify the automated segmentation tool best suited for radiation therapy planning in Korean cancer patients.

## 2. Materials and Methods

### 2.1. Study Design

This study was approved by the Institutional Review Board (IRB) of Yonsei Cancer Center (IRB No. 1-2022-0047) and conducted in accordance with international ethical standards for clinical research. As the study used retrospective data, the requirement for informed consent was waived by the IRB. We retrospectively collected treatment data, including CT images and associated radiotherapy structures, in Digital Imaging and Communications in Medicine (DICOM) format. These data were gathered from a cohort of 1200 consecutive cancer patients who underwent radiotherapy at Yonsei Cancer Center between January 2019 and June 2022.

The primary objective of this study was to evaluate whether tools developed using population-specific datasets are more clinically applicable and effective for particular cohorts. We compared the clinical efficacy and segmentation performance of two automated contouring tools, OncoStudio (OncoSoft Inc., Seoul, Republic of Korea) and Protégé AI (MIM Software Inc., Cleveland, OH, USA), in the context of radiotherapy planning. Protégé AI is based on the U-Net architecture, widely used for segmentation, which processes input images into segmentation masks. OncoStudio utilizes a modified U-Net architecture featuring long skip connections, instance normalization, parameterized RELU activations, and a Squeeze-and-Excitation (SE) block to enhance performance. The version of Protégé AI used in this study was developed using datasets that included diverse populations from various countries. In contrast, the version of OncoStudio used in this study was developed using a dataset primarily composed of Korean patients.

To achieve our research objective, we first utilized manual contours of OARs that had been delineated and approved by radiation oncologists for actual treatment as the reference standard. For each patient’s CT dataset, auto-contours of all relevant OARs were generated using OncoStudio and Contour Protégé AI, resulting in two sets of auto-contours per case. The accuracy of these auto-contours was assessed by comparison with manual contours using well-established quantitative metrics. In addition, the clinical applicability of each tool was evaluated through subjective assessments and feedback from experts from the Department of Radiation Oncology, including radiation oncologists, residents, and radiation therapists, who routinely use these systems in practice. This dual approach—quantitative analysis and clinician feedback—provided a comprehensive evaluation of the strengths and limitations of both segmentation tools in a real-world clinical setting.

### 2.2. Evaluation Metrics and Statistical Analysis

Several quantitative metrics were employed to assess the similarity between the manual contours and the auto-contours generated by OncoStudio and Contour Protégé AI. The data from 1200 patients were categorized on the basis of anatomical location, with 200 cases each from head and neck organs, thoracic organs of male patients, thoracic organs of female patients, abdominal organs, pelvic organs of male patients, and pelvic organs of female patients for detailed analysis. The Dice similarity coefficient (DSC) was used as the primary measure of overlap between the two sets of contours—with values ranging from 0 to 1—with higher DSC values indicating greater overlap. The DSC was calculated for both volumetric overlap (volumetric DSC) and surface overlap (surface DSC) using the following formula [20]:

$$DSC=\frac{2(V_{1}\;\cap \;V_{2})}{V_{1}+V_{2}},$$

where

- *V*_1_ represents the volume of the manual contour,
- *V*_2_ represents the volume of the auto-contour.

In addition to DSC, the mean surface distance (MSD) was calculated to measure the average distance between the surfaces of the manual and auto-contours. The MSD evaluates how well the surfaces of the two contour sets align, providing insight into the overall geometric accuracy of the segmentation. A lower MSD value indicates better alignment between the surfaces. The formula for the MSD is as follows [21]:

$$MSD=\frac{1}{\left|S_{1}\right|}\sum_{x\in S_{1}}\underset{y\in S_{2}}{\min}d\left(x,y\right)+\frac{1}{\left|S_{2}\right|}\sum_{y\in S_{2}}\underset{x\in S_{1}}{\min}d\left(y,x\right),$$

where

- *S*_1_ and *S*_2_ represent the surfaces of the manual and auto-contours, respectively,
- *d*(x,y) is the Euclidean distance between a point *x* on *S*_1_ and the closest point y on *S*_2_.

This bidirectional calculation ensures that both surface sets are equally considered.

To account for extreme outliers in contour discrepancies, we calculated the 95% Hausdorff distance (HD), which measures the distance at which 95% of the points on one surface lie within a certain threshold of the other surface. Unlike traditional HD, which measures the maximum deviation between surfaces, 95% HD excludes the most extreme 5% of outlier points, providing a more robust indicator of contour agreement. A lower 95% HD indicates closer correspondence between the two surfaces. The formula for 95% HD is as follows [22]:

$$HD95=\max\left(\underset{x\in S_{1}}{\sup}\underset{y\in S_{2}}{\inf}d\left(x,y\right),\underset{y\in S_{2}}{\sup}\underset{x\in S_{1}}{\inf}d\left(x,y\right)\right)$$

where

- *d*(x,y) is the Euclidean distance between a point *x* on *S*_1_ and the closest point y on *S*_2_ (auto-contours).

For the statistical comparison between the two auto-contouring tools, independent *t*-tests were performed for each metric (DSC, MSD, and 95% HD) to assess significant differences in performance. The differences in each metric for individual OARs were visualized using boxplots generated with GraphPad Prism software (version 10.3.1, GraphPad Software Inc.). All the statistical analyses were conducted using SPSS software (version 27, IBM Corp., Armonk, NY, USA), with statistical significance set at *p* < 0.05.

### 2.3. Qualitative Comparative Evaluation via Turing Test

A Turing test [23] was conducted with clinician feedback across various anatomical regions (head and neck organs, thoracic organs of male patients, thoracic organs of female patients, abdominal organs, pelvic organs of male patients, and pelvic organs of female patients) to evaluate the clinical feasibility of deep learning-based auto-segmentation tools. The survey was based on CT images and contours from patients involved in the study and was administered through Google Forms, allowing participants to complete it online. The survey comprised three parts: (1) questions on professional background, experience with radiotherapy, and opinions on deep learning-based auto-segmentation; (2) blind identification of whether contours were created manually or by auto-segmentation tools and error severity assessment; and (3) pairwise comparisons of contours generated by OncoStudio, Protégé AI, and manual contouring. Importantly, all contours were evaluated in a fully blinded manner, with participants unaware of whether the contours were created manually or by AI tools, and also blinded to which specific AI tool generated the auto-contours. This survey was conducted with 10 participants, including four board-certified radiation oncologists, one resident, and five radiation therapists. The survey comprised 108 questions and took approximately 30 min to complete. The full details are available in Supplementary Material S1.

## 3. Results

**Figure 1:**
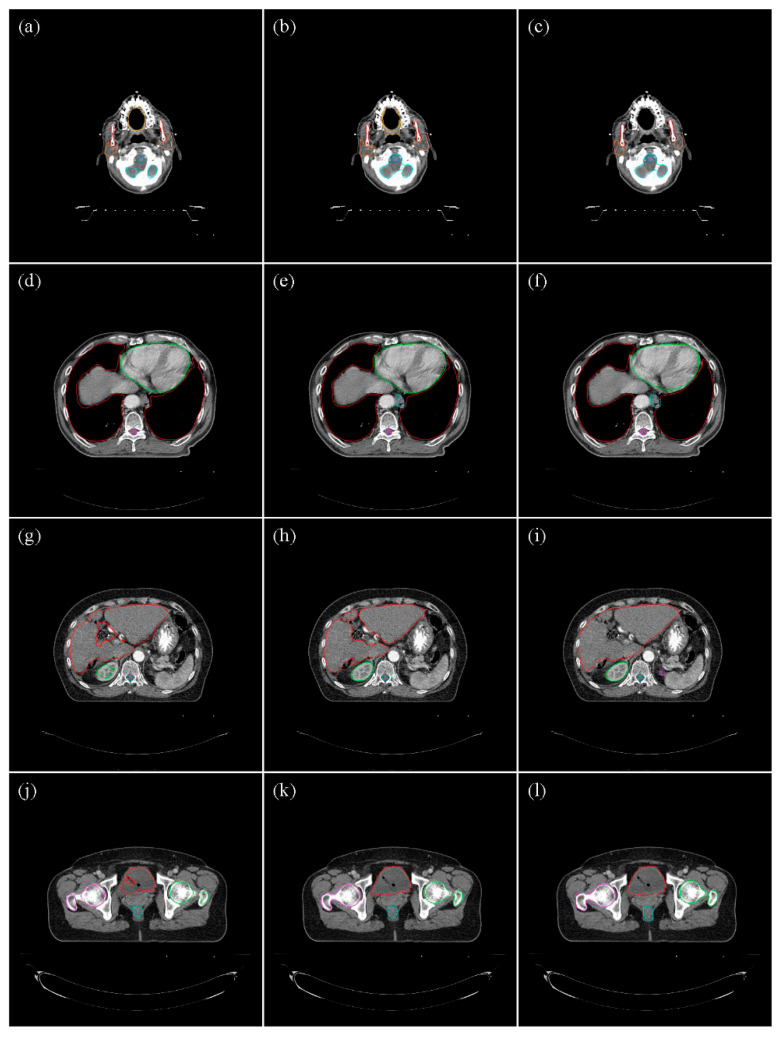
Examples of Auto-Contours from Protégé AI, OncoStudio, and Manual Contours Across Body Regions. The figure above displays representative contouring results for each body-region. The first row presents a head and neck case, the second row features a thoracic case, the third row illustrates an abdominal case, and the fourth row depicts a pelvic case. The left column (**a**,**d**,**g**,**j**) represents Protégé AI, the middle column (**b**,**e**,**h**,**k**) represents OncoStudio, and the right column (**c**,**f**,**i**,**l**) represents manual contouring.

This study evaluated 20 OARs in the head and neck region, four in the thoracic region of male patients, five in the female thoracic region, four in the abdomen, and four each in the male and female pelvic regions. OncoStudio and Protégé AI auto-contours were compared to manual contours using DSC, MSD, and 95% HD values (Figure 1). The survey participants had varying levels of experience: 10% with less than 1 year, 20% with 1–5 years, 40% with 6–10 years, and 30% with more than 10 years of experience. Regarding deep learning knowledge, 20% of the participants had only media-level familiarity, 40% understood the fundamentals, 30% had conducted research, and 10% had clinical experience with deep learning. Most participants (90%) agreed that auto-segmentation could be feasibly implemented in clinical practice and provide significant benefits, while 10% believed that further validation was required.

### 3.1. Head and Neck Organs

**Figure 2:**
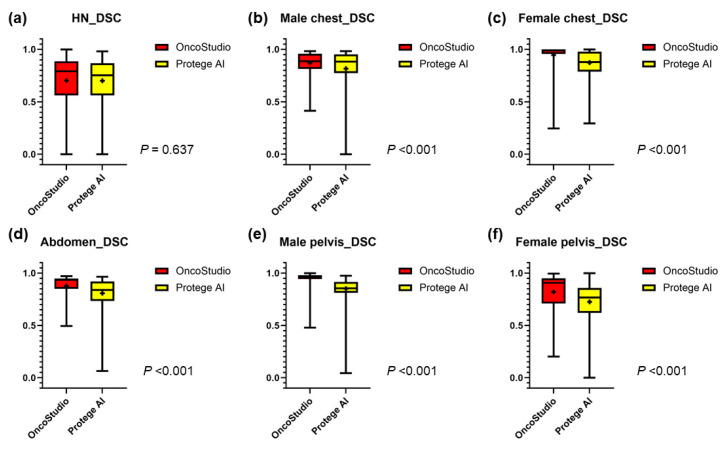
Boxplot for Dice similarity coefficient (DSC) comparison between manual contours and auto-contours generated by each auto-segmentation tool for organs at risk (OARs) in each different region: (**a**) head and neck organs, (**b**) thoracic organs of male patients, (**c**) thoracic organs of female patients, (**d**) abdominal organs, (**e**) pelvic organs of male patients, and (**f**) pelvic organs of female patients. The boxes indicate the interquartile ranges (IQRs, 25th to 75th percentiles), with the horizontal lines inside the boxes representing the median values. The whiskers extend to the minimum and maximum values, and the plus signs (“+”) denote the mean values for each group. *p*-values indicate the statistical significance of the differences between OncoStudio and Protégé AI, as determined by a *t*-test.

**Figure 3:**
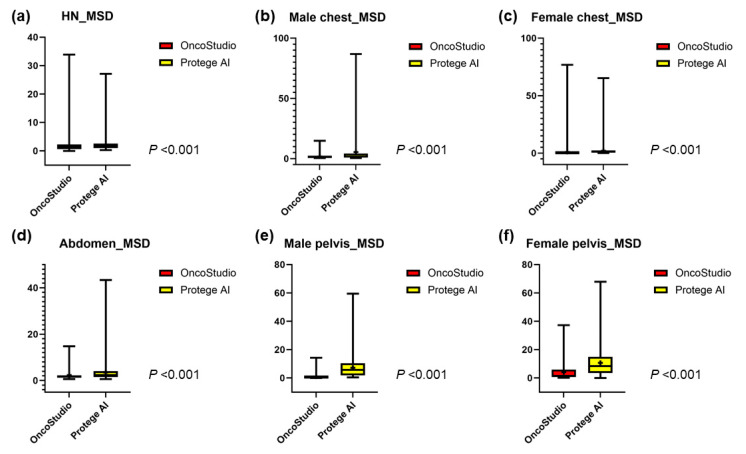
Boxplot for mean surface distance (MSD) comparison between manual contours and auto-contours generated by each auto-segmentation tool for OARs in each different region: (**a**) head and neck organs, (**b**) thoracic organs of male patients, (**c**) thoracic organs of female patients, (**d**) abdominal organs, (**e**) pelvic organs of male patients, and (**f**) pelvic organs of female patients. The boxes indicate the IQRs (25th to 75th percentiles), with the horizontal lines inside the boxes representing the median values. The whiskers extend to the minimum and maximum values, and the plus signs (“+”) denote the mean values for each group. *p*-values indicate the statistical significance of the differences between OncoStudio and Protégé AI, as determined by a *t*-test.

**Figure 4:**
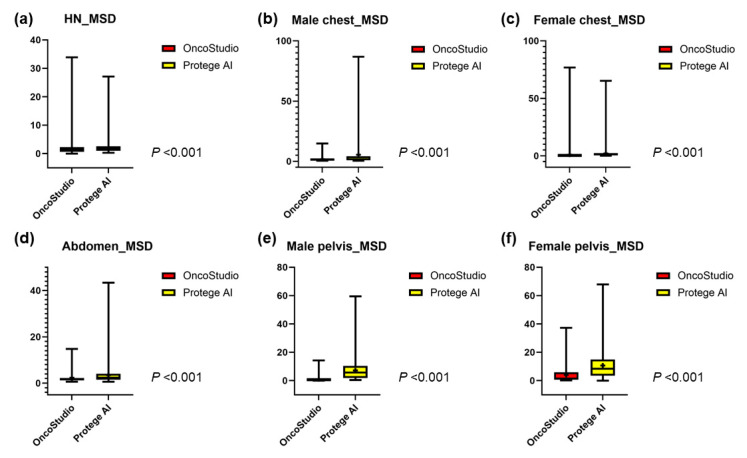
Boxplot for 95% Hausdorff distance (HD) comparison between manual contours and auto-contours generated by each auto-segmentation tool for OARs in each different region: (**a**) head and neck organs, (**b**) thoracic organs of male patients, (**c**) thoracic organs of female patients, (**d**) abdominal organs, (**e**) pelvic organs of male patients, and (**f**) pelvic organs of female patients. The boxes indicate the IQR (25th to 75th percentiles), with the horizontal lines inside the boxes representing the median values. The whiskers extend to the minimum and maximum values, and the plus signs (“+”) denote the mean values for each group. *p*-values indicate the statistical significance of the differences between OncoStudio and Protégé AI, as determined by a *t*-test.

In the head and neck region, the mean DSCs for all OARs between OncoStudio and Protégé AI were similar (0.70 vs. 0.70, *p* = 0.637); however, OncoStudio demonstrated significantly lower MSD (1.75 vs. 2.15, *p* < 0.001) and 95% HD values (5.00 vs. 6.60, *p* < 0.001), indicating better contour alignment (Figure 2a, Figure 3a and Figure 4a). Most OARs had a DSC above 0.70; however, smaller and more complex structures, such as the cochlea (Cochlea_L: 0.37 vs. 0.44, *p* < 0.001; Cochlea_R: 0.41 vs. 0.47, *p* < 0.001), lens (Lens_L: 0.32 vs. 0.59, *p* < 0.001; Lens_R: 0.27 vs. 0.54, *p* < 0.001), optic chiasm (0.50 vs. 0.40, *p* < 0.001), and optic nerve (Optic nerve_L: 0.64 vs. 0.56, *p* < 0.001), showed lower agreement with manual contours for both tools. OncoStudio outperformed Protégé AI in 14 of 16 OARs, with notable improvements in the submandibular glands (SMG_L: 0.82 vs. 0.76, *p* < 0.001; SMG_R: 0.81 vs. 0.71, *p* < 0.001) and parotid glands (Parotid gland_L: 0.81 vs. 0.80, *p* = 0.051; Parotid gland_R: 0.84 vs. 0.82, *p* = 0.001). Protégé AI performed better in the oral cavity (0.75 vs. 0.71, *p* < 0.001) and spinal cord (0.83 vs. 0.80, *p* < 0.001), but OncoStudio consistently showed superior MSD (submandibular glands: SMG_L: 1.45 vs. 1.82, *p* = 0.001; SMG_R: 1.58 vs. 2.50, *p* < 0.001) and 95% HD values (parotid glands: Parotid gland_L: 6.37 vs. 6.40, *p* = 0.928; Parotid gland_R: 5.18 vs. 5.75, *p* = 0.024) across most OARs (Table 1, Figure S1).

On the basis of the results of the online survey assessing clinical feasibility, in Sample 1, clinicians identified both OncoStudio and Protégé AI contours as AI-generated contours, whereas only 30% recognized the manual contours as AI-generated contours. Eighty percent of the clinicians reported that OncoStudio’s contours required corrections, whereas 90% of Protégé AI contours did. The difference was more pronounced in Sample 2, where corrections were necessary for 40% of the OncoStudio contours than for 90% of the Protégé AI contours. Although manual contours were preferred over both auto-contouring tools, OncoStudio was favored over Protégé AI when the two were compared directly, with 70% vs. 10% in Sample 1 and 60% vs. 10% in Sample 2. These findings suggest that OncoStudio offers superior performance, with fewer corrections and higher clinician satisfaction, compared to Protégé AI (Table S1).

### 3.2. Thoracic Organs

In male patients, OncoStudio achieved significantly higher DSC values across all OARs than Protégé AI (0.87 vs. 0.82, *p* < 0.001), with lower MSD (1.79 vs. 5.42, *p* < 0.001) and 95% HD (6.67 vs. 20.32, *p* < 0.001) (Figure 2b, Figure 3b and Figure 4b). Notably, OncoStudio outperformed Protégé AI in the esophagus (DSC: 0.79 vs. 0.55, *p* < 0.001; MSD: 1.61 vs. 15.72, *p* < 0.001; 95% HD: 5.18 vs. 59.62, *p* < 0.001) and in the heart (DSC: 0.91 vs. 0.89, *p* < 0.001; MSD: 3.50 vs. 4.00, *p* = 0.006), although Protégé AI had a slightly higher DSC for the spinal cord (DSC: 0.85 vs. 0.82, *p* < 0.001; MSD: 0.97 vs. 1.29, *p* = 0.002; 95% HD: 2.57 vs. 4.19, *p* = 0.043). In female patients, OncoStudio consistently outperformed Protégé AI, achieving higher DSC values across all OARs (0.95 vs. 0.87, *p* < 0.001), including the right lung (0.99 vs. 0.98, *p* < 0.001) and the heart (0.98 vs. 0.88, *p* < 0.001; MSD: 0.94 vs. 3.63, *p* < 0.001; 95% HD: 4.48 vs. 15.13, *p* < 0.001). OncoStudio also showed lower MSD (1.04 vs. 2.02, *p* < 0.001) and 95% HD values (4.59 vs. 8.55, *p* < 0.001), except for the esophagus (Figure 2c, Figure 3c, Figure 4c and Figure S2, Table 2).

The results of the online survey assessing clinical feasibility were as follows. For male patients, OncoStudio required fewer corrections than Protégé AI in both Sample 1 (40% vs. 90%) and Sample 2 (10% vs. 70%). Direct comparison of contours revealed that manual contours were generally preferred over auto-contours. However, OncoStudio was favored over Protégé AI in Sample 1 (70% vs. 10%) and Sample 2 (50% vs. 30%). For female patients, OncoStudio required fewer corrections in Sample 1 (50% vs. 90%) and Sample 2 (30% vs. 80%). Direct comparisons revealed a preference for OncoStudio in Sample 1 (70% vs. 30%) and an overwhelming preference in Sample 2 (100% vs. 0%), whereas OncoStudio’s contours were equally favored with manual contours (Table S2).

### 3.3. Abdominal Organs

In the abdominal organs, OncoStudio yielded significantly greater DSC values across all OARs than Protégé AI (0.88 vs. 0.81, *p* < 0.001), with lower MSD values (2.34 vs. 3.50, *p* < 0.001), although no significant difference was observed in the 95% HD values (12.04 vs. 11.18, *p* = 0.222) (Figure 2d, Figure 3d and Figure 4d). OncoStudio performed particularly well in the liver and kidneys, achieving DSC values of 0.93 vs. 0.89 (*p* < 0.001) and 0.94 vs. 0.80 (*p* < 0.001), respectively. Protégé AI outperformed OncoStudio only in the spinal cord (DSC: 0.74 vs. 0.70, *p* < 0.001; MSD: 1.94 vs. 3.96, *p* < 0.001; 95% HD: 6.94 vs. 22.42, *p* < 0.001). Additionally, OncoStudio had lower MSD values, particularly in the kidneys (Kidney_R: 1.34 vs. 4.11, *p* < 0.001; Kidney_L: 1.48 vs. 4.20, *p* < 0.001) (Figure 3 and Figure S3, Table 3).

On the basis of the results of the clinical feasibility assessment, for Sample 1, 80% of the clinicians indicated that OncoStudio’s contours required corrections, whereas 100% required corrections for Protégé AI, with most indicating a need for major corrections in the latter. In Sample 2, 50% of OncoStudio’s contours required minor corrections, whereas 70% of Protégé AI’s contours needed corrections. Comparing the auto-contours directly, OncoStudio was preferred over Protégé AI in Sample 1 (80% vs. 20%) and Sample 2 (70% vs. 30%), indicating higher clinician satisfaction with OncoStudio’s performance (Table S3).

### 3.4. Pelvic Organs

For the pelvic organs, OncoStudio outperformed Protégé AI across all the male OARs, achieving a mean DSC of 0.95 vs. 0.85 for Protégé AI (*p* < 0.001). The MSD (0.97 vs. 7.20, *p* < 0.001) and 95% HD values (5.59 vs. 47.46, *p* < 0.001) were also significantly lower, indicating higher accuracy (Figure 2e, Figure 3e and Figure 4e). For female patients, OncoStudio demonstrated higher DSC values across all OARs (mean DSC: 0.82 vs. 0.73, *p* < 0.001), except for the anorectum, where both tools had lower DSC scores (0.68 vs. 0.60, *p* < 0.001). The MSD (4.07 vs. 10.64, *p* < 0.001) and 95% HD (18.05 vs. 54.12, *p* < 0.001) results followed the same trend, further demonstrating OncoStudio’s superiority (Figure 2f, Figure 3f, Figure 4f and Figure S4, Table 4).

On the basis of the results of the clinical feasibility assessment for male patients, 30% of the clinicians indicated that OncoStudio’s contours required only minor corrections in Sample 1, whereas 90% did for Protégé AI, with 70% requiring major corrections. In Sample 2, both tools required more corrections, with 80% for OncoStudio and 90% for Protégé AI. Direct comparison favored manual contours; however, among the auto-contours, OncoStudio was preferred for Sample 1 (80% vs. 20%) and Sample 2 (40% vs. 10%). A similar trend was observed in female patients, where OncoStudio required fewer corrections than Protégé AI (20% vs. 100% in Sample 1 and 70% vs. 100% in Sample 2). Manual contours were consistently preferred, followed by OncoStudio, with Protégé AI rated lowest (Table S4).

## 4. Discussion

This study highlights the superior performance of OncoStudio, an AI-based auto-segmentation tool developed specifically for Korean patients, over Protégé AI, a globally developed tool. OncoStudio consistently outperformed Protégé AI across most OARs, particularly in the head and neck, thoracic, abdominal, and pelvic regions. The significantly higher DSC values and lower MSD and 95% HD observed with OncoStudio suggest that it offers more precise segmentation tailored to Korean anatomical structures. This finding emphasizes the importance of population-specific development in medical AI tools, as anatomical variability can significantly impact segmentation accuracy and clinical utility.

Although OncoStudio performed excellently for most OARs, there was no significant difference in the DSC values between OncoStudio and Protégé AI across all head and neck organs, and for some specific OARs, OncoStudio showed lower concordance. Protégé AI demonstrated better performance in structures such as the oral cavity, cochlea, lens, and spinal cord. However, OncoStudio still achieved DSC values above 0.7 for the oral cavity and spinal cord, which is considered clinically acceptable, making the difference in these regions not necessarily indicative of clinical inferiority. Furthermore, the overall trend of lower segmentation accuracy in smaller, anatomically complex structures, such as the cochlea and lens, diminished the ability to assess differences fully between the tools for head and neck organs. OncoStudio’s lower segmentation accuracy in smaller, anatomically complex structures, such as the cochlea and lens, contrasts with its superior performance in larger, well-defined OARs, such as the submandibular and parotid glands. These results underscore the known limitations of both AI tools in handling smaller, intricate anatomical structures, where segmentation accuracy often declines. This highlights the need for further refinement of auto-segmentation models to improve performance in these more challenging structures. OncoStudio’s lower segmentation accuracy in smaller, anatomically complex structures, such as the cochlea and lens, contrasts with its superior performance in larger, well-defined OARs, such as the submandibular and parotid glands. These findings align with those of previous studies [24,25], which reported the limitations of auto--segmentation systems in contouring smaller, intricate structures, especially when visualized using CT rather than MRI.

Recent advancements in AI for radiotherapy have significantly enhanced precision, efficiency, and adaptability in clinical practice. AI-driven auto-segmentation, especially when U-Net convolutional neural networks are utilized, has become pivotal in minimizing interobserver variability and improving contouring accuracy [26,27]. AI is also being increasingly integrated into automated treatment planning [28,29], where knowledge-based models optimize the dose distribution for more personalized care. In online adaptive radiotherapy, AI facilitates real-time motion tracking and plan adaptation, reducing on-couch time while ensuring precise radiation delivery [30,31,32,33]. Innovations such as pseudo-CT generation further streamline these adaptive processes [34,35,36,37,38]. In addition to segmentation and planning, AI has made strides in clinical decision-making, from personalized treatment prescriptions to risk stratification and response assessment [39]. However, challenges related to generalizability and reproducibility, particularly across diverse patient populations, remain and underscore the necessity for ongoing research.

With auto-segmentation tools becoming increasingly integral to radiation oncology, enhancing precision and efficiency in radiotherapy planning, our center has led a series of studies assessing the clinical utility of these technologies. In 2019 [15], atlas-based auto-segmentation in the head and neck region was shown to reduce manual workload, although some corrections were required. In 2020 [16], deep learning-based auto-segmentation demonstrated superior accuracy compared with atlas-based methods, particularly in more complex anatomical regions. In 2021, studies on Oncosoft, a deep learning tool for breast cancer, showed marked improvements in target volume delineation [17]. Research in 2022 focused on synthetic contrast CT for cardiac substructures [18] and brain metastases [19], further enhancing segmentation accuracy. In 2023, auto-segmentation for cervical cancer OARs and target volumes showed promise in streamlining pelvic planning [40]. Our most recent study in 2024, which focused on deep learning-based auto-contouring for breast cancer, further optimized accuracy in clinical applications [41]. Building on these foundations, the current study aims to address the gaps in population-specific segmentation models, particularly those focused on Korean patients, and contributes to the global evolution of these technologies.

A notable gap in the current literature is the lack of attention given to anatomical and physiological variations between different ethnic groups. Most globally developed tools, such as Protégé AI, are designed for broad applicability; however, they may not account for subtle anatomical nuances that can vary across populations. This study addresses this gap by demonstrating that OncoStudio, tailored specifically to the Korean population, consistently outperforms a globally developed tool across various organ regions. These findings underscore the importance of developing auto-segmentation tools that account for ethnic and anatomical diversity to reduce manual corrections and improve clinical outcomes in radiation therapy. As this field evolves, population-specific auto-segmentation tools, such as OncoStudio, could play a critical role in enhancing radiotherapy planning precision for diverse patient populations.

The Turing test applied in this study offers valuable insights into the clinical utility of auto-segmentation tools by assessing clinicians’ ability to distinguish between autogenerated and manual contours. The clinicians in our study overwhelmingly favored OncoStudio over Protégé AI, reflecting its closer alignment with manual contours and fewer required corrections. This highlights the test’s relevance for evaluating technical performance and understanding how well a tool integrates into clinical workflows. However, its subjective nature can introduce variability, as individual preferences and experience may influence assessments. Unlike objective metrics, such as DSC, MSD, and 95% HD, which provide quantitative analysis, the Turing test offers a qualitative perspective on clinical applicability [42,43,44]. All contours were evaluated in a fully blinded manner, minimizing bias in clinician assessments. Despite this, the relatively small number of participants may limit the diversity of feedback, and future research with a larger and more varied sample will improve robustness. Nevertheless, compared with studies focusing solely on quantitative measures, our approach combines clinician feedback with objective data, offering a more comprehensive evaluation of auto-segmentation performance. Future research should fundamentally integrate subjective and quantitative assessments for a balanced analysis of tool effectiveness.

Despite these encouraging results, the study has several limitations. First, the reference manual contours were delineated by a single expert radiation oncologist. While this ensured consistency, it does not account for the inter-observer variability typically seen in clinical practice. Previous study [45] has shown that deep learning models trained on single-expert data can achieve comparable accuracy to those trained on multi-expert data; however, future studies should include contours from multiple clinicians to enhance generalizability. Second, while all OARs should be considered in radiotherapy planning, this study was limited to specific OARs, excluding challenging structures, such as the stomach, duodenum, and bowels, due to the limited development of segmentation tools for these regions. Future research should include a broader range of OARs to better reflect clinical practice. Third, the study focused exclusively on a Korean patient cohort, and further validation across more diverse populations is necessary. Fourth, the study focused solely on OAR segmentation, excluding tumor segmentation, which is essential for radiotherapy planning. Accurate tumor segmentation, especially in cases with complex or irregularly shaped tumors, is critical for precise dose delivery and treatment efficacy. Additionally, we were unable to assess the computational resources or time required for auto-segmentation, which are crucial for understanding its impact on clinical workflow. Finally, variations in image acquisition protocols and CT scanner models may affect segmentation performance, and future studies should explore these variables.

Compared to previous studies on auto-segmentation tools, our study stands out due to its large-scale evaluation across a diverse set of OARs and the inclusion of a population-specific AI tool. Many earlier studies have focused on smaller datasets or single anatomical regions, limiting their generalizability. In contrast, our research involved 1200 cases across various anatomical regions, offering a more comprehensive evaluation of AI tools in clinical practice. Additionally, while other studies have primarily used global AI tools, our comparison between Protégé AI and OncoStudio, a population-specific tool tailored for Korean patients, provides valuable insights into the advantages of customized models. We also incorporated a multi-faceted evaluation approach by employing both quantitative metrics and a Turing test with clinician feedback, which allowed for a more holistic comparison of the tools’ clinical utility. These factors contribute to the broader applicability and clinical relevance of our findings.

## 5. Conclusions

This study demonstrated that OncoStudio, a population-specific auto-segmentation tool, offers superior segmentation accuracy over Protégé AI in Korean patients. Our findings highlight the necessity of developing medical AI tools that are tailored to specific populations, particularly in radiation therapy, where precise anatomical delineation is crucial for optimal treatment outcomes. Future research should focus on validating these findings in more ethnically and anatomically diverse populations to ensure broader applicability of the tools across different clinical environments. Additionally, integrating tumor segmentation into auto-segmentation tools is a critical next step. Tumor segmentation, especially for complex or irregularly shaped tumors, is essential for accurate dose delivery in radiotherapy. Further studies should also explore the tools’ scalability in clinical workflows, assessing performance across varying imaging protocols and hardware setups.

## Figures and Tables

**Table 1:** Comparison of similarity metrics between manual contours used in real treatments and auto-contours generated by each auto-segmentation tool for organs at risk (OARs) in the head and neck region.

OAR	DSC			MSD			95% HD		
	OncoStudio	Protégé AI	*p*-Value	OncoStudio	Protégé AI	*p*-Value	OncoStudio	Protégé AI	*p*-Value
Mandible	0.89	0.87	0.058	1.45	1.65	0.457	6.26	6.57	0.775
SMG_L	0.82	0.76	<0.001	1.45	1.82	0.001	5.26	5.71	0.280
SMG_R	0.81	0.71	<0.001	1.58	2.50	<0.001	5.73	7.43	0.050
Brain	0.99	0.97	<0.001	0.36	1.30	<0.001	1.92	4.28	<0.001
Brainstem	0.83	0.82	0.400	1.96	2.05	0.646	5.58	5.95	0.537
Oral cavity	0.71	0.75	<0.001	5.11	5.17	0.790	14.90	14.50	0.511
Cochlea_L	0.37	0.44	<0.001	2.46	2.45	0.948	5.03	5.84	<0.001
Cochlea_R	0.41	0.47	<0.001	2.71	2.52	0.598	5.61	5.98	0.646
Eye_L	0.92	0.90	0.007	0.68	0.78	0.026	2.21	2.58	<0.001
Eye_R	0.91	0.90	0.053	0.68	0.78	0.070	2.23	2.53	0.006
Thyroid gland	0.84	0.76	<0.001	0.73	1.69	<0.001	2.43	5.69	<0.001
Larynx	0.76	0.59	<0.001	2.93	5.54	<0.001	7.03	15.19	<0.001
Lens_L	0.32	0.59	<0.001	2.00	1.19	<0.001	3.50	2.80	<0.001
Lens_R	0.27	0.54	<0.001	2.22	1.32	<0.001	3.72	2.96	<0.001
Optic chiasm	0.50	0.40	<0.001	1.46	2.28	<0.001	4.62	5.96	<0.001
Optic nerve_L	0.64	0.56	<0.001	1.06	2.41	<0.001	4.33	12.21	<0.001
Optic nerve_R	0.63	0.55	<0.001	1.34	2.35	<0.001	5.23	10.97	<0.001
Parotid gland_L	0.81	0.80	0.051	1.96	2.12	0.031	6.37	6.40	0.928
Parotid gland_R	0.84	0.82	0.001	1.68	1.91	<0.001	5.18	5.75	0.024
Spinal cord	0.80	0.83	<0.001	1.12	1.05	0.056	2.67	2.47	0.011

Abbreviations: DSC, Dice similarity coefficient; MSD, mean surface distance; HD, Hausdorff distance; OAR, organ at risk; SMG, submandibular gland; L, left; R, right; AI, artificial intelligence.

**Table 2:** Comparison of similarity metrics between manual contours used in real treatments and auto-contours generated by each auto-segmentation tool for OARs in the thoracic region.

	DSC			MSD			95% HD		
OAR	OncoStudio	Protégé AI	*p*-Value	OncoStudio	Protégé AI	*p*-Value	OncoStudio	Protégé AI	*p*-Value
Male patients									
Lungs	0.97	0.97	<0.001	0.95	1.00	0.122	3.27	3.65	0.017
Heart	0.91	0.89	<0.001	3.50	4.00	0.006	14.91	15.33	0.602
Spinal cord	0.82	0.85	<0.001	1.29	0.97	0.002	4.19	2.57	0.043
Esophagus	0.79	0.55	<0.001	1.61	15.72	<0.001	5.18	59.62	<0.001
Female patients									
Lung_R	0.99	0.98	<0.001	0.25	0.56	<0.001	0.80	2.05	<0.001
Lung_L	0.99	0.98	<0.001	0.21	0.62	<0.001	0.72	2.21	<0.001
Heart	0.98	0.88	<0.001	0.94	3.63	<0.001	4.48	15.13	<0.001
Spinal cord	0.94	0.77	<0.001	1.19	2.92	0.009	4.94	12.53	0.010
Esophagus	0.83	0.76	<0.001	2.61	2.39	0.661	12.01	10.85	0.664

Abbreviations: DSC, Dice similarity coefficient; MSD, mean surface distance; HD, Hausdorff distance; OAR, organ at risk; SMG, submandibular gland; L, left; R, right; AI, artificial intelligence.

**Table 3:** Comparison of similarity metrics between manual contours used in real treatments and auto-contours generated by each auto-segmentation tool for OARs in the abdominal region.

	DSC			MSD			95% HD		
OAR	OncoStudio	Protégé AI	*p*-Value	OncoStudio	Protégé AI	*p*-Value	OncoStudio	Protégé AI	*p*-Value
Liver	0.93	0.89	<0.001	2.56	3.77	<0.001	11.59	14.37	0.005
Kidney_R	0.94	0.80	<0.001	1.34	4.11	<0.001	6.64	11.75	<0.001
Kidney_L	0.94	0.79	<0.001	1.48	4.20	<0.001	7.52	11.64	<0.001
Spinal_Cord	0.70	0.74	<0.001	3.96	1.94	<0.001	22.42	6.94	<0.001

Abbreviations: DSC, Dice similarity coefficient; MSD, mean surface distance; HD, Hausdorff distance; OAR, organ at risk; SMG, submandibular gland; L, left; R, right; AI, artificial intelligence.

**Table 4:** Comparison of similarity metrics between manual contours used in real treatments and auto-contours generated by each auto-segmentation tool for OARs in the pelvic region.

	DSC			MSD			95% HD		
Organ	OncoStudio	Protégé AI	*p*-Value	OncoStudio	Protégé AI	*p*-Value	OncoStudio	Protégé AI	*p*-Value
Male patients									
Bladder	0.97	0.92	<0.001	0.57	1.75	<0.001	3.10	5.97	<0.001
Femur_L	0.96	0.86	<0.001	0.75	9.15	<0.001	4.30	68.39	<0.001
Femur_R	0.96	0.81	<0.001	0.80	13.06	<0.001	4.39	90.91	<0.001
Anorectum	0.93	0.82	<0.001	1.77	4.83	<0.001	10.56	24.59	<0.001
Female patients									
Bladder	0.91	0.81	<0.001	1.72	4.16	<0.001	6.57	15.66	<0.001
Femur_L	0.85	0.76	<0.001	3.37	11.82	<0.001	15.12	69.11	<0.001
Femur_R	0.85	0.74	<0.001	3.45	14.19	<0.001	15.76	80.87	<0.001
Anorectum	0.68	0.60	<0.001	7.96	12.30	<0.001	36.11	50.22	<0.001

Abbreviations: DSC, Dice similarity coefficient; MSD, mean surface distance; HD, Hausdorff distance; OAR, organ at risk; SMG, submandibular gland; L, left; R, right; AI, artificial intelligence.

## Data Availability

The data presented in this study are available upon request from the corresponding author.

## Supplementary Materials

The following supporting information can be downloaded at: https://www.mdpi.com/article/10.3390/cancers16213670/s1, Figure S1. Boxplots comparing OncoStudio and Protégé AI for organs-at-risk in the head and neck region using (a) Dice Similarity Coefficient (DSC), (b) Mean Surface Distance (MSD), and (c) 95% Hausdorff Distance (95% HD). Figure S2. Boxplots comparing OncoStudio and Protégé AI for thoracic organs in male and female patients using (a,d) Dice Similarity Coefficient (DSC), (b,e) Mean Surface Distance (MSD), and (c,f) 95% Hausdorff Distance (95% HD). (a–c) display results for male patients, and (d–f) display results for female patients. Figure S3. Boxplots comparing OncoStudio and Protégé AI for organs-at-risk in the abdominal region using (a) Dice Similarity Coefficient (DSC), (b) Mean Surface Distance (MSD), and (c) 95% Hausdorff Distance (95% HD). Figure S4. Boxplots comparing OncoStudio and Protégé AI for pelvic organs in male and female patients using (a, d) Dice Similarity Coefficient (DSC), (b,e) Mean Surface Distance (MSD), and (c,f) 95% Hausdorff Distance (95% HD). (a–c) display results for male patients, and (d–f) display results for female patients. Table S1. Clinician evaluation results of head and neck organ contours: Comparison of manual contours and auto-contours generated by OncoStudio and Protégé AI using subjective clinician assessments and contour preference. Table S2. Clinician evaluation results of thoracic contours: Comparison of manual contours and auto-contours generated by OncoStudio and Protégé AI using subjective clinician assessments and contour preference. Table S3. Clinician evaluation results of abdominal contours: Comparison of manual contours and auto-contours generated by OncoStudio and Protégé AI using subjective clinician assessments and contour preference. Table S4. Clinician evaluation results of pelvis contours: Comparison of manual contours and auto-contours generated by OncoStudio and Protégé AI using subjective clinician assessments and contour preference. Supplementary Material S1: online survey.
