# Supplementary material for: Automated Organ Segmentation for Radiation Therapy: A Comparative Analysis of AI-Based Tools Versus Manual Contouring in Korean Cancer Patients
Source: Cancers (Basel). 2024 Oct 30;16(21):3670. doi: 10.3390/cancers16213670 (PMC11544936; doi:10.3390/cancers16213670)

Supplementary Figures

Figure S1. Boxplots comparing OncoStudio and Protégé AI for organs-at-risk in the head and neck region using (a) Dice Similarity Coefficient (DSC), (b) Mean Surface Distance (MSD), and (c) 95% Hausdorff Distance (95% HD).

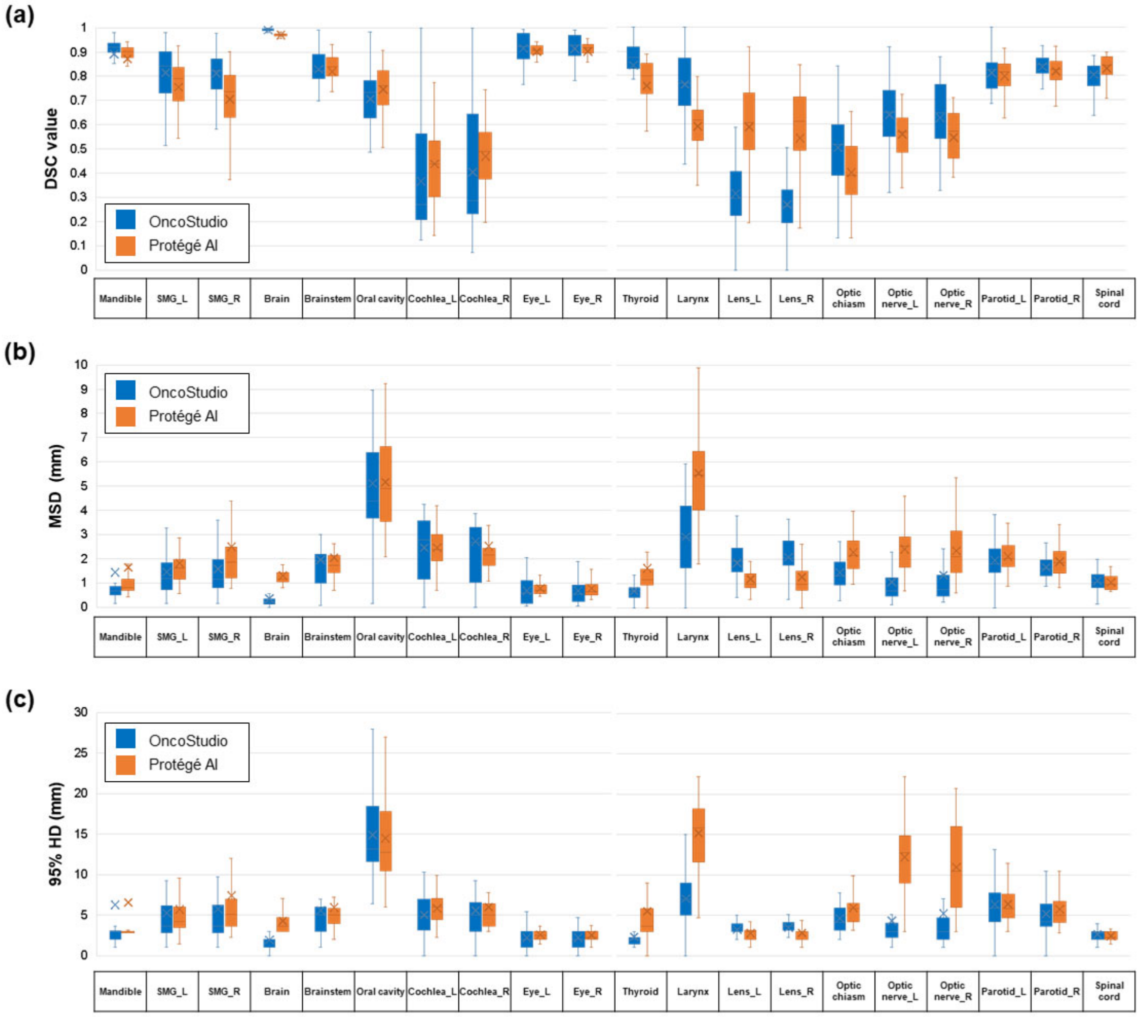

Figure S2. Boxplots comparing OncoStudio and Protégé AI for thoracic organs in male and female patients using (a, d) Dice Similarity Coefficient (DSC), (b, e) Mean Surface Distance (MSD), and (c, f) 95% Hausdorff Distance (95% HD). (a-c) display results for male patients, and (d-f) display results for female patients.

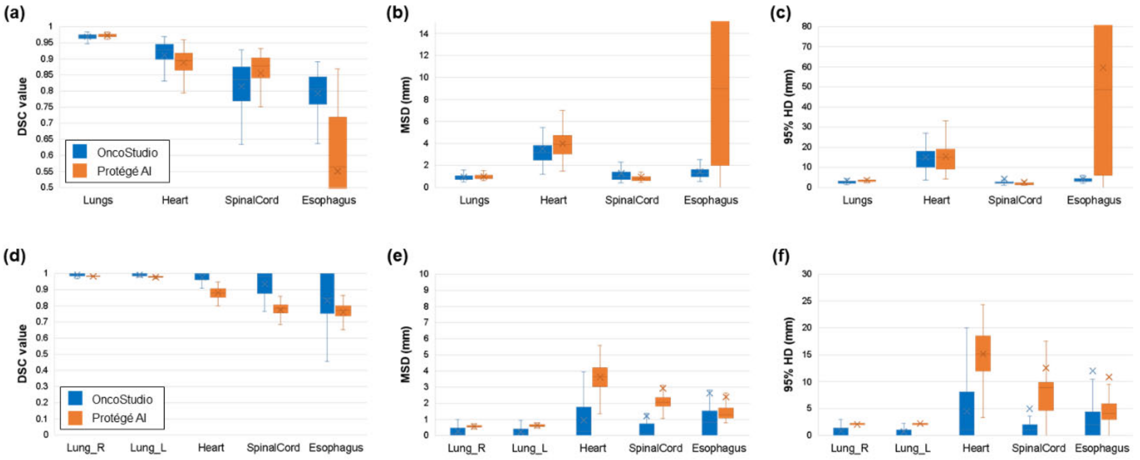

Figure S3. Boxplots comparing OncoStudio and Protégé AI for organs-at-risk in the abdominal region using (a) Dice Similarity Coefficient (DSC), (b) Mean Surface Distance (MSD), and (c) 95% Hausdorff Distance (95% HD).

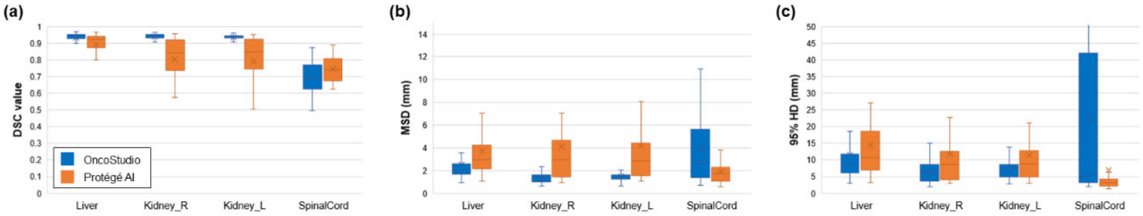

Figure S4. Boxplots comparing OncoStudio and Protégé AI for pelvic organs in male and female patients using (a, d) Dice Similarity Coefficient (DSC), (b, e) Mean Surface Distance (MSD), and (c, f) 95% Hausdorff Distance (95% HD). (a-c) display results for male patients, and (d-f) display results for female patients.

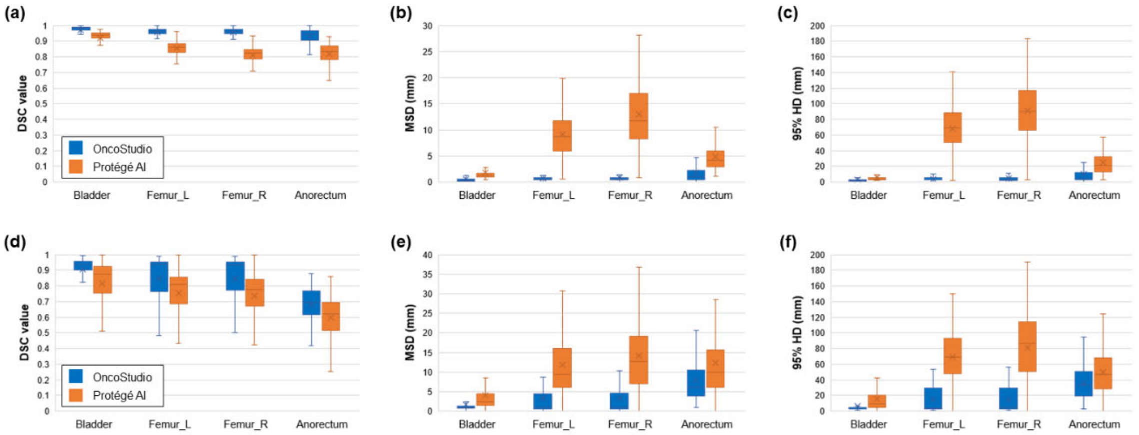

Supplement: Supplementary file 1 [file cancers-16-03670-s001.zip › Supplementary figures.pdf]
