# Supplementary material for: Automated Organ Segmentation for Radiation Therapy: A Comparative Analysis of AI-Based Tools Versus Manual Contouring in Korean Cancer Patients
Source: Cancers (Basel). 2024 Oct 30;16(21):3670. doi: 10.3390/cancers16213670 (PMC11544936; doi:10.3390/cancers16213670)
Supplement: Supplementary file 1 [file cancers-16-03670-s001.zip › Supplementary Material S1_online survey.pdf]

A. Basic Questions

1. **Occupation**:
    - a. Radiation oncologist (board-certified specialist)
    - b. Radiation oncologist (resident in training)
    - c. Radiation therapist

2. **Experience in Radiation Therapy**:
    - a. Less than 1 year
    - b. 1–5 years
    - c. 6–10 years
    - d. More than 10 years

3. **Level of Education on Deep Learning**:
    - a. I only know what is covered in the media.
    - b. I understand the general concept.
    - c. I have conducted research using deep learning.
    - d. I have applied it in actual clinical work.

4. **Opinion on Deep Learning-Based Auto-Segmentation**:
    - a. It is a temporary research trend and not ideal for long-term research.
    - b. Useful for research purposes, but clinical efficacy needs more validation.
    - c. Clinical application seems difficult, and it is mostly for research purposes. It may be possible to implement clinically, but it would not have a significant impact.
    - d. It is feasible for clinical implementation and will be significantly beneficial.

[Both Part 1 (B) and Part 2 (C) were conducted separately for each anatomical region: head and neck organs, thoracic organs of male patients, thoracic organs of female patients, abdominal organs, pelvic organs of male patients, and pelvic organs of female patients.]

B. Part 1: Contour Evaluation

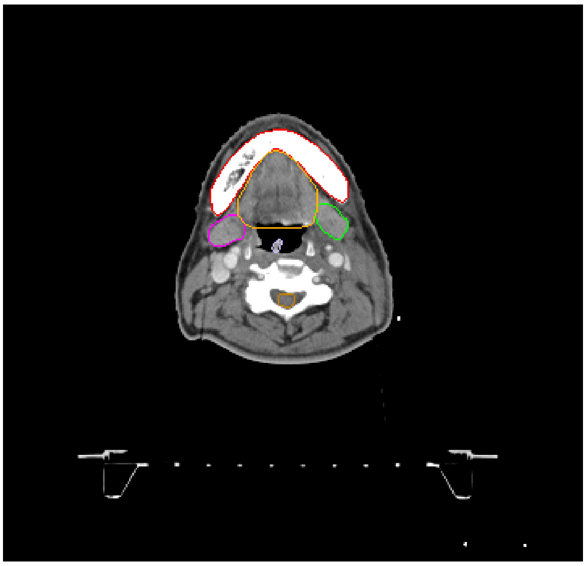

(All contours for organs-at-risk (OARs) on each CT slice are provided as video clips.)

1. **How do you think this contour was generated?**
   - a. By computer (AI)
   - b. By human

2. **What do you think about the contour's validity for clinical application?**
   - a. Needs major corrections (major errors present)
   - b. Needs minor corrections (minor errors that require slight adjustments)
   - c. Needs no correction (minor errors that are clinically insignificant)
   - d. Needs no correction (highly accurate)

C. Part 2: Comparative Contour Evaluation

Part 2-1: Comparison between Oncosoft and Manual

Part 2-2: Comparison between Oncosoft and Protégé AI

Part 2-3: Comparison between Protégé AI and Manual

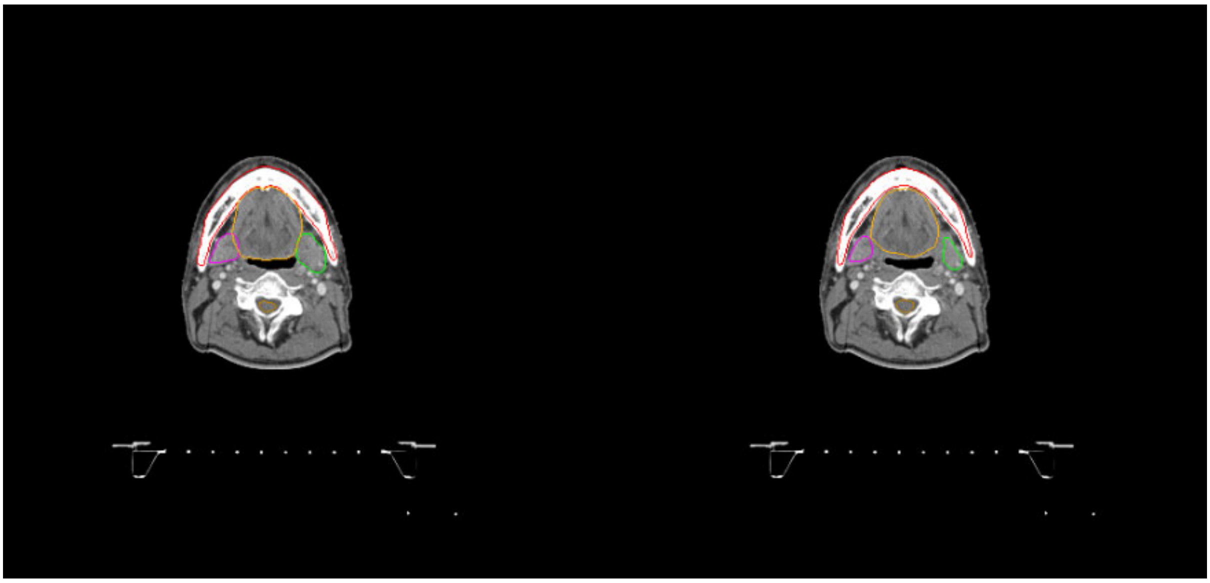

(All contours for organs-at-risk (OARs) on each CT slice are provided as video clips.)

1. **Which contour do you prefer?**

   - a. Left
   - b. Right
   - c. Prefer neither
