# Supplementary material for: Automated Organ Segmentation for Radiation Therapy: A Comparative Analysis of AI-Based Tools Versus Manual Contouring in Korean Cancer Patients
Source: Cancers (Basel). 2024 Oct 30;16(21):3670. doi: 10.3390/cancers16213670 (PMC11544936; doi:10.3390/cancers16213670)
Supplement: Supplementary file 1 [file cancers-16-03670-s001.zip › Supplementary Tables.pdf]

Supplementary Tables

Table S1. Clinician evaluation results of head and neck organ contours: Comparison of manual contours and auto-contours generated by OncoStudio and Protégé AI using subjective clinician assessments and contour preference.

Part 1: Contour Evaluation

Patient	Contour Type	Q1		Q2			
		By computer (%)	By human (%)	Needs major correction (%)	Needs minor correction (%)	Needs no correction (Clinically Insignificant Error) (%)	Needs no correction (highly accurate) (%)
**Head and Neck Organs**							
Patient 1	OncoStudio	100	0	30	50	20	0
	Protégé AI	100	0	20	70	10	0
	Manual	30	70	0	60	20	20
Patient 2	OncoStudio	50	50	10	30	60	0
	Protégé AI	90	10	50	40	10	0
	Manual	30	70	0	70	10	20

Part 2: Comparative Contour Evaluation

Patient	Comparison	Q1	
		Contour preference	%
**Head and Neck Organs**			
Patient 1	OncoStudio vs. Manual	OncoStudio	20
		Manual	80
		Neither	0
	Protégé AI vs. Manual	Protégé AI	30
		Manual	60
		Neither	10
	OncoStudio vs. Protégé AI	OncoStudio	70
		Protégé AI	10

		Neither	20
Patient 2	OncoStudio vs. Manual	OncoStudio	20
		Manual	80
		Neither	0
	Protégé AI vs. Manual	Protégé AI	10
		Manual	90
		Neither	0
	OncoStudio vs. Protégé AI	OncoStudio	60
		Protégé AI	10
		Neither	30

Table S2. Clinician evaluation results of thoracic contours: Comparison of manual contours and auto-contours generated by OncoStudio and Protégé AI using subjective clinician assessments and contour preference

		Part 1: Contour Evaluation					
**Patient**	**Contour Type**	**Q1**		**Q2**			
		**By computer (%)**	**By human (%)**	**Needs major correction (%)**	**Needs minor correction (%)**	**Needs no correction (Clinically Insignificant Error) (%)**	**Needs no correction (highly accurate) (%)**
**Thoracic organs (male)**							
Patient 1	OncoStudio	70	30	10	30	40	20
	Protégé AI	90	10	30	60	10	0
	Manual	0	100	0	10	70	20
Patient 2	OncoStudio	80	20	0	10	70	20
	Protégé AI	80	20	20	50	20	10
	Manual	0	100	0	20	50	30
**Thoracic organs (female)**							
Patient 1	OncoStudio	80	20	0	50	50	0
	Protégé AI	80	20	20	70	10	0
	Manual	0	100	0	70	30	0
Patient 2	OncoStudio	50	50	0	30	50	20
	Protégé AI	100	0	30	50	20	0
	Manual	0	100	0	50	30	20

	Part 2: Comparative Contour Evaluation		
**Patient**	**Comparison**	**Q1**	
		**Contour preference**	**%**
**Thoracic organs (male)**			
Patient 1	OncoStudio vs. Manual	OncoStudio	0
		Manual	100

		Neither	0
	Protégé AI vs. Manual	Protégé AI	10
		Manual	90
		Neither	0
	OncoStudio vs. Protégé AI	OncoStudio	70
		Protégé AI	10
		Neither	20
Patient 2	OncoStudio vs. Manual	OncoStudio	0
		Manual	100
		Neither	0
	Protégé AI vs. Manual	Protégé AI	0
		Manual	100
		Neither	0
	OncoStudio vs. Protégé AI	OncoStudio	50
		Protégé AI	30
		Neither	20
**Thoracic organs (female)**			
Patient 1	OncoStudio vs. Manual	OncoStudio	30
		Manual	70
		Neither	0
	Protégé AI vs. Manual	Protégé AI	20
		Manual	80
		Neither	0
	OncoStudio vs. Protégé AI	OncoStudio	70
		Protégé AI	30
		Neither	0
Patient 2	OncoStudio vs. Manual	OncoStudio	50
		Manual	50
		Neither	0

Protégé AI vs. Manual	Protégé AI	20
	Manual	80
	Neither	0
OncoStudio vs. Protégé AI	OncoStudio	100
	Protégé AI	0
	Neither	0

Table S3. Clinician evaluation results of abdominal contours: Comparison of manual contours and auto-contours generated by OncoStudio and Protégé AI using subjective clinician assessments and contour preference

		Part 1: Contour Evaluation					
Patient	Contour Type	Q1		Q2			
		By computer (%)	By human (%)	Needs major correction (%)	Needs minor correction (%)	Needs no correction (Clinically Insignificant Error) (%)	Needs no correction (highly accurate) (%)
**Abdominal Organs**							
Patient 1	OncoStudio	100	0	30	50	20	0
	Protégé AI	100	0	80	20	0	0
	Manual	20	80	0	80	20	0
Patient 2	OncoStudio	70	30	0	50	50	0
	Protégé AI	80	20	20	50	20	10
	Manual	20	80	0	20	80	0

	Part 2: Comparative Contour Evaluation		
Patient	Comparison	Q1	
		Contour preference	%
**Abdominal Organs**			
Patient 1	OncoStudio vs. Manual	OncoStudio	20
		Manual	30
		Neither	50
	Protégé AI vs. Manual	Protégé AI	30
		Manual	70
		Neither	0
	OncoStudio vs. Protégé AI	OncoStudio	80
		Protégé AI	20
		Neither	0

Patient 2	OncoStudio vs. Manual	OncoStudio	20
		Manual	80
		Neither	0
	Protégé AI vs. Manual	Protégé AI	30
		Manual	70
		Neither	0
	OncoStudio vs. Protégé AI	OncoStudio	70
		Protégé AI	30
		Neither	0

Table S4. Clinician evaluation results of pelvis contours: Comparison of manual contours and auto-contours generated by OncoStudio and Protégé AI using subjective clinician assessments and contour preference

Part 1: Contour Evaluation

Patient	Contour Type	Q1		Q2			
		By computer (%)	By human (%)	Needs major correction (%)	Needs minor correction (%)	Needs no correction (Clinically Insignificant Error) (%)	Needs no correction (highly accurate) (%)
**Pelvic organs (male)**							
Patient 1	OncoStudio	80	20	0	30	70	0
	Protégé AI	100	0	70	20	10	0
	Manual	50	50	0	10	70	20
Patient 2	OncoStudio	100	0	70	10	20	0
	Protégé AI	100	0	70	20	10	0
	Manual	50	50	0	10	70	20
**Pelvic organs (female)**							
Patient 1	OncoStudio	70	30	0	20	50	30
	Protégé AI	100	0	30	70	0	0
	Manual	70	30	0	30	50	20
Patient 2	OncoStudio	80	20	20	50	30	0
	Protégé AI	100	0	50	50	0	0
	Manual	70	30	20	50	30	0

Part 2: Comparative Contour Evaluation

Patient	Comparison	Q1	
		Contour preference	%
**Pelvic organs (male)**			
Patient 1	OncoStudio vs. Manual	OncoStudio	30
		Manual	70

		Neither	0
	Protégé AI vs. Manual	Protégé AI	0
		Manual	100
		Neither	0
	OncoStudio vs. Protégé AI	OncoStudio	80
		Protégé AI	20
		Neither	0
Patient 2	OncoStudio vs. Manual	OncoStudio	20
		Manual	80
		Neither	0
	Protégé AI vs. Manual	Protégé AI	20
		Manual	80
		Neither	0
	OncoStudio vs. Protégé AI	OncoStudio	40
		Protégé AI	10
		Neither	50
**Pelvic organs (female)**			
Patient 1	OncoStudio vs. Manual	OncoStudio	20
		Manual	80
		Neither	0
	Protégé AI vs. Manual	Protégé AI	0
		Manual	100
		Neither	0
	OncoStudio vs. Protégé AI	OncoStudio	70
		Protégé AI	10
		Neither	20
Patient 2	OncoStudio vs. Manual	OncoStudio	30
		Manual	70
		Neither	0

Protégé AI vs. Manual	Protégé AI	0
	Manual	100
	Neither	0
OncoStudio vs. Protégé AI	OncoStudio	80
	Protégé AI	20
	Neither	0
